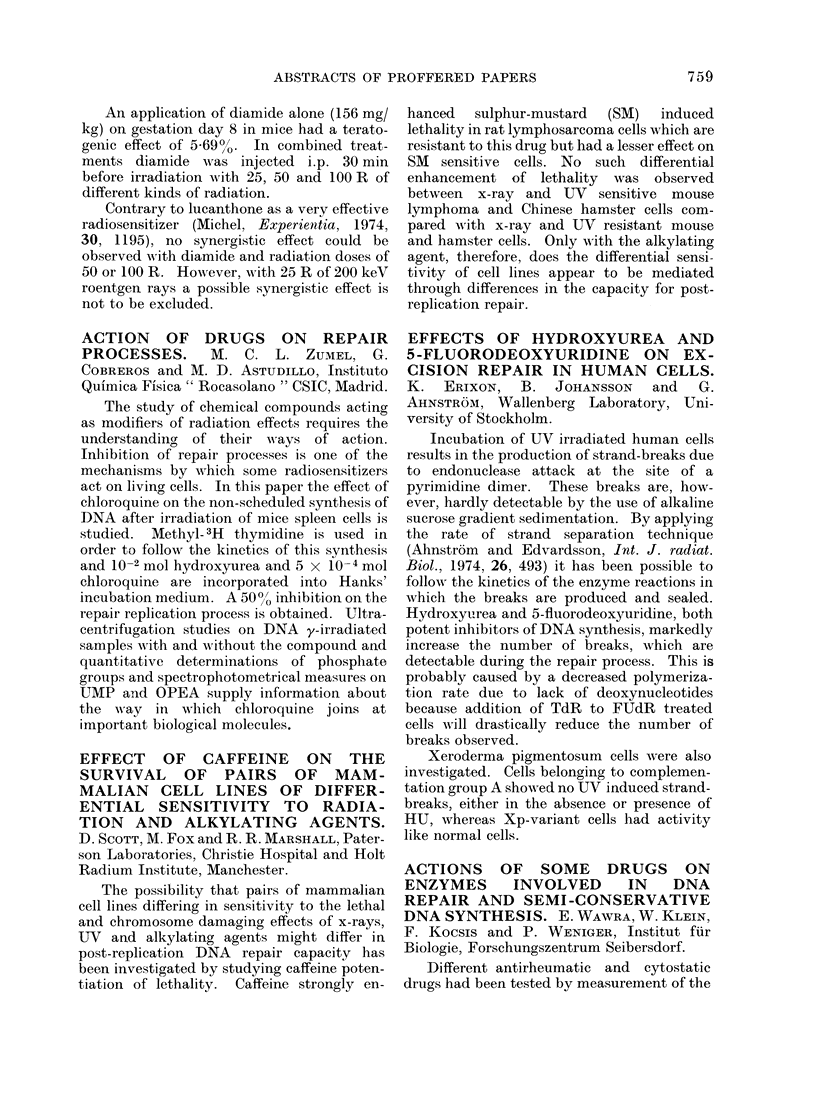# Proceedings: Effect on caffeine on the survival of pairs of mammalian cell lines of differential sensitivity to radiation and alkylating agents.

**DOI:** 10.1038/bjc.1975.316

**Published:** 1975-12

**Authors:** D. Scott, M. Fox, R. P. Marshall


**EFFECT OF CAFFEINE ON THE SURVIVAL OF PAIRS OF MAMMALIAN CELL LINES OF DIFFERENTIAL SENSITIVITY TO RADIATION AND ALKYLATING AGENTS.** D. Scott, M. Fox and R. R. Marshall, Paterson Laboratories, Christie Hospital and Holt Radium Institute, Manchester.

The possibility that pairs of mammalian cell lines differing in sensitivity to the lethal and chromosome damaging effects of x-rays, UV and alkylating agents might differ in post-replication DNA repair capacity has been investigated by studying caffeine potentiation of lethality. Caffeine strongly enhanced sulphur-mustard (SM) induced lethality in rat lymphosarcoma cells which are resistant to this drug but had a lesser effect on SM sensitive cells. No such differential enhancement of lethality was observed between x-ray and UV sensitive mouse lymphoma and Chinese hamster cells compared with x-ray and UV resistant mouse and hamster cells. Only with the alkylating agent, therefore, does the differential sensitivity of cell lines appear to be mediated through differences in the capacity for post-replication repair.